# Effects of a Low Dose of Fish Oil on Inflammatory Markers of Brazilian HIV-Infected Adults on Antiretroviral Therapy: A Randomized, Parallel, Placebo-Controlled Trial

**DOI:** 10.3390/nu7085294

**Published:** 2015-08-05

**Authors:** Julicristie M. Oliveira, Patrícia H. C. Rondó, Lourdes R. A. V. Lima, Elizabeth S. Fortuna, John S. Yudkin

**Affiliations:** 1 School of Applied Sciences, University of Campinas, Sao Paulo 13484-350, Brazil; 2 Department of Nutrition, School of Public Health, University of Sao Paulo, Sao Paulo 01246-904, Brazil; E-Mail: phcrondo@usp.br; 3 Laboratory of Immunology, Adolfo Lutz Institute, Sao Paulo 13036-225, Brazil; E-Mail: lourlima@uol.com.br; 4 University College London, London WC1H 0EG, UK; E-Mail: j.yudkin@ucl.ac.uk

**Keywords:** HIV infection, AIDS, metabolic syndrome, inflammation, C reactive protein, fibrinogen, factor VIII, interleukin 6, interleukin 1-beta, tumor necrosis factor-alpha

## Abstract

Background: The benefits of antiretroviral therapy for HIV-infected subjects have been limited by an increased risk of metabolic and cardiovascular diseases. The objective of this study was to assess the effects of a low dose of marine omega-3 fatty acids on inflammatory marker concentrations in HIV-infected subjects under antiretroviral therapy (ART). Methods: This was a randomized, parallel, placebo-controlled trial that investigated the effects of 3 g fish oil/day (540 mg of eicosapentaenoic acid—EPA plus 360 mg of docosahexaenoic acid—DHA) or 3 g soy oil/day (placebo) for 24 weeks in 83 male and non-pregnant female HIV-infected adults on ART. Results: There were no differences between groups for the measures at baseline. Multilevel analyses revealed no statistically significant relationship between the longitudinal changes in high sensitivity-C reactive protein (hs-CRP) (Wald Chi2 = 0.17, *p* = 0.918), fibrinogen (Wald Chi2 = 3.82, *p* = 0.148), and factor VIII (Wald Chi2 = 5.25, *p* = 0.073) with fish oil. No significant changes in interleukin-6 (IL6), interleukin-1 beta (IL1-beta) and tumor necrosis factor-alpha (TNF-alpha) serum concentrations were observed with fish oil supplements for 12 weeks. Conclusions: Compared to placebo, a low dose of 900 mg omega-3 fatty acids (EPA plus DHA) in fish oil capsules did not change hs-CRP, fibrinogen, factor VIII, IL6, IL1-beta and TNF-alpha serum concentrations in HIV-infected subjects on ART. Further investigations should consider the assessment of more sensitive inflammatory markers or higher doses to evaluate the effects of marine omega-3 fatty acids in this population. Registered at the Nederlands Trial Register, Identifier no. NTR1798.

## 1. Introduction

The benefits of antiretroviral therapy (ART) on morbidity and mortality of HIV-infected subjects have been limited by the increased risk of the metabolic syndrome [1] and cardiovascular diseases [2]. Although the basic mechanisms of these alterations are still not completely elucidated, they are probably secondary to a synergistic effect between HIV infection, certain antiretroviral drugs, genetic, and environmental factors [3].

There is increasing evidence supporting the role of inflammation in the metabolic syndrome and cardiovascular (CV) diseases and some biomarkers of chronic mild inflammation are emerging as novel CV risk factors [4,5,6,7]. Concomitantly, clinical trials have assessed the effects of marine omega-3 fatty acids as anti-inflammatory agents in subjects with the metabolic syndrome and cardiovascular diseases [8]. We have found only one publication that has investigated the impact of omega-3 fatty acids on the inflammatory profile of HIV-subjects on ART [9]. Thus, the aim of this paper is to answer the following question: Can a low dose of marine omega-3 fatty acids reduce or prevent the increases in concentrations of C reactive protein (hs-CRP), fibrinogen, factor VIII, interleukin-6 (IL-6), interleukin-beta (IL-beta), and tumor necrosis factor-alpha (TNF-alpha) in HIV-infected subjects on antiretroviral treatment? The rationale for choosing these inflammatory markers was related to their association with cardiovascular disease (hs-CRP, fibrinogen, VIII factor) and with components of the metabolic syndrome (IL-6, IL-beta, TNF-alpha).

## 2. Materials and Methods

This was a randomized, parallel, placebo-controlled trial registered at the Nederlands Trial Register, Identifier no. NTR1798. The study was developed at an HIV/AIDS Care Centre in São Paulo city, Brazil, affiliated to the Medical School, University of São Paulo.

The sample size was calculated to detect a mean difference of 0.40 mg/L in hs-CRP concentrations between groups (power: 95%, alpha: 5%), and adjusted for an estimated follow-up loss rate of 25%, resulting in 120 subjects (~60 per group).

From January to October 2009, 768 subjects’ medical records were evaluated for eligibility. The inclusion criteria were: Men or non-pregnant women, between 19 and 64 years old, on at least three months of stable ART, blood triglycerides >100 mg/dL (1.13 mmoL/L), LDL-Cholesterol (LDL-C) concentrations <160 mg/dL (4.14 mmoL/L), fasting glucose <126 mg/dL (7.0 mmoL/L), without symptoms of any comorbidity, and not currently using any nutritional supplement, anticoagulant, hypoglycaemic, or lipid lowering drugs. From May 2009 to October 2010, 215 subjects were invited to participate in the trial by phone or during their routine health care at the Centre by one of the authors, JMO, and 120 subjects gave their consent. The study was approved by the Ethics in Research Committees of the Public Health School and Medical School Hospital, University of Sao Paulo, Brazil.

The subjects were divided into two groups using a randomized number table generated in Excel^®^. The intervention group received 3 g fish oil/day (*n* = 62, 3 capsules weighting 1 g each, total 540 mg of eicosapentaenoic acid—EPA plus 360 mg of docosahexaenoic acid—DHA) or 3 g of soy oil/day (*n* = 58, 3 placebo capsules with 1 g each) for 24 weeks. The supplements were provided by Relthy^®^ Pharmacy Company (Indaiatuba, Sao Paulo, Brazil). The fish oil and placebo capsules were almost identical with a slight difference in color. Although the study was designed to be double-masked, during the protocol some subjects detected a fishy taste so we decided not to consider this a masked trial. Fish oil capsule compliance and potential side effects were monitored during follow up. The subjects received three bottles of supplement at baseline and at 12 weeks, and were asked to return these, empty or not, at the 12 weeks and 24 weeks visit, respectively. The adherence was calculated by the ratio of the numbers of capsules provided to those returned.

A questionnaire was administered by JMO and other field staff to obtain socioeconomic, demographic and health information. Weight and height (in duplicate) were obtained with a portable Tanita^®^ balance and a CMS Weighing Equipment^®^ anthropometer, as weight in kg/(height in meters)^2^. Body circumferences (waist, hip and neck) were measured (in duplicate) with a TBW^®^ non-elastic metric tape.

Serum hs-CRP, fibrinogen, and factor VIII were determined at the Central Laboratory of the Medical School Hospital, University of Sao Paulo. Subjects’ blood samples were collected at baseline, and at the 12 week and 24 week visit, after 12 h of fasting. Serum hs-CRP was analyzed by immunoturbidimetry (CardioPhase^®^ hs-CRP, Siemens, Berlin, Germany). Fibrinogen was determined by modified Clauss method and factor VIII was estimated based on the activated partial thromboplastin time (APTT) (BCS System^®^ XP, Siemens, Berlin, Germany). The lymphocyte T CD4+ and CD8+ were analyzed by flow cytometry (FACSCalibur^®^, BD Biosciences, San Jose, CA, USA) and HIV viral load was determined by directed quantification of HIV-1 RNA in plasma (Bayer System^®^ 340—bDNA Analyzer).

Serum IL-6, IL-1beta and TNF-alpha were evaluated by ELISA (enzyme-linked immunosorbent assay with kits from R&D Systems, MN, USA, Quantikine) at the Immunology Laboratory, Adolfo Lutz Institute. Serum hs-CRP was determined by immunoturbidimetry (CardioPhase^®^ hs-CRP, Siemens), lymphocytes T CD4+ and CD8+ were analyzed by flow cytometry (FACSCalibur^®^, BD Biosciences) at the Central Laboratory of the Medical School Hospital, University of Sao Paulo. For these inflammatory markers, measures were obtained only at the baseline and at the 12th week visit.

Between the baseline and 12 weeks, 26 subjects dropped out, and between 12 and 24 weeks, a further 12. During the analysis, data from 11 subjects who started lipid-lowering medications and those with hs-CRP >10 mg/L were excluded, because of the possibility of an intercurrent infection. Thus, our final sample was 83 subjects with at least two measurements: Baseline and 12th week visit. Among these 83 subjects, we had three measurements: Baseline, 12th week visit, and 24th week visit for 71 subjects. Further design details are in Oliveira *et al.* [10], where a flow diagram of patients recruited and the reasons for dropping out can be found.

The primary outcomes were not normally distributed; thus, the data are expressed as median and inter-quartile range. The non-parametric Mann-Whitney test was used for inter-group comparisons at baseline. The same test was applied to assess changes in serum IL-6, IL-1beta and TNF-alpha after 12 weeks of intervention. For serum hs-CRP, fibrinogen and VIII factor, multilevel analyses with random effects were carried out to assess the longitudinal fish oil effects. The differences were considered statistically significant based on a p value of <0.05 regarding Mann-Whitney Tests and multilevel analyses. Data were analyzed with the software Stata 9 (Stata Corporation, College Station, TX, USA).

## 3. Results

Subject characteristics are described in Table 1. Eighty three men and women with a mean of age 43.0 ± 6.8 years (range 26–61) were included. The mean CD4+ cell count was 604 ± 315 cells/mm^3^, and was comparable between groups. The majority of subjects were male, single/divorced/widowed, had normal BMI and were on protease inhibitors. Adherence to supplements was 85.1% (7.7%) and 83.7% (13.5%) in the fish oil and placebo groups, respectively. Some subjects from the fish oil group experienced transient episodes of diarrhoea (*n* = 4), abdominal pain (*n* = 2), loss of appetite (*n* = 1), heartburn (*n* = 2), and headache (*n* = 3). Only two subjects from the placebo group experienced transient episodes of diarrhoea (*n* = 1), and heartburn (*n* = 1). All these side effects developed early during the study and did not result in patients stopping the supplements. No effects of the fish oil were noted on CD4+ count, CD4+/CD8+ ratio or viral load.

There were no statistically significant differences between groups at baseline for primary outcomes (Table 2 and Table 3). No significant relationships were observed between the administration of fish oil supplements and changes in concentrations of inflammatory markers (Table 3 and Table 4).

**Table 1:** Characteristics of the subjects.

Characteristics/Groups	Fish Oil	Placebo	Total
*N* (%)	43 (51.8%)	40 (48.2%)	83 (100%)
Age, mean (SD)	43.1 (7.4)	42.8 (6.3)	43.0 (6.8)
**Gender, *n* (%)**			
Male	33 (76.7%)	31 (77.5%)	64 (77.1%)
Female	10 (23.3%)	9 (22.5%)	19 (22.9%)
**Marital status**			
Married/stable relationship	19 (44.2%)	18 (45.0%)	37 (44.6%)
Single/divorced/widowed	24 (55.8%)	22 (55.5%)	46 (55.4%)
**Socio-economic factors, mean (SD)**			
*Per capita* income/month	2.5 (1.7)	3.3 (3.0)	2.9 (2.4)
Years of study	13.2 (4.3)	12.3 (3.8)	12.7 (4.1)
**Life style characteristics, *n* (%)**			
Smoking	14 (32.6%)	21 (52.5%)	35 (42.2%)
Alcohol intake	29 (67.4%)	26 (65.0%)	55 (66%)
**Nutritional status, mean (SD)**			
BMI	24.8 (3.6)	23.2 (3.8)	24.0 (3.8)
Waist circumference (cm)	89.2 (10.2)	85.0 (10.8)	87.2 (10.6)
Waist/hip ratio	0.92 (0.08)	0.90 (0.08)	0.91 (0.08)
Neck circumference (cm)	38.4 (3.6)	36.8 ( 3.5)	37.7 (3.6)
**HIV/Aids parameters, mean (SD)**			
CD4+ cell count (cells/mm^3^) ^#^	591.8 (259.6)	616.2 (366.9)	603.8 (315.4)
CD4+/CD8+ ratio	0.93 (0.69)	0.74 (0.41)	0.84 (0.58)
HIV viral load ^##^	40.3 (182.5)	65.8 (244.7)	53.0 (214.8)
Years of infection	10.3 (5.7)	10.9 (5.0)	10.6 (5.4)
Years of ART	8.3 (4.1)	9.2 (3.5)	8.7 (3.9)
**Current ART, *n* (%)**			
2 NRTI + 1 NNRTI	20 (46.5%)	15 (37.5%)	35 (42.2%)
2 NRTI + 1 PI	6 (14.0%)	2 (5.0%)	8 (9.6%)
2 NRTI + 2 PI	13 (30.2%)	14 (35.0%)	27 (32.5%)
Other combinations	4 (9.3%)	9 (22.5%)	13 (15.7%)
Use of PI	22 (51.2%)	25 (62.5%)	47 (56.6%)
Use of NNRTI	21 (48.8%)	16 (40.0%)	37 (44.5%)
Supplement compliance, mean (SD) **	85.1% (7.7%)	83.7% (13.5%)	84.4% (11.0%)

* In Brazilian Minimum Wages—BMW (one BMW = 306 US dollar); ^#^ two missing values; ^##^ one outlier value excluded; SD: standard deviation; ART: antiretroviral therapy; NRTI: nucleoside reverse transcriptase inhibitor; NNRTI: non-nucleoside reverse transcriptase inhibitor; PI: protease inhibitor; ** 10 missing values.

**Table 2:** Primary endpoints—C Reactive Protein, Fibrinogen, Factor VIII.

	Fish Oil		Placebo	
	*N*	Median (IQR)	*N*	Median (IQR)
**High sensitivity C-reactive protein (mg/L)**				
Baseline *	43	2.34 (0.74–3.75)	40	1.68 (0.76–3.23)
12th week	42	1.93 (0.79–3.15)	36	1.40 (0.87–3.13)
24th week	31	2.36 (0.65–3.52)	35	2.10 (0.94–3.40)
**Fibrinogen (mg/dL)**				
Baseline **	43	266 (236–364)	39	323 (260–415)
12th week	42	275 (234–349)	35	320 (251–405)
24th week	31	262 (233–346)	34	306 (245–388)
**VIII factor (%)**				
Baseline ***	43	92 (65–110)	38	83 (69–113)
12th week	42	83 (61–106)	33	78 (51–97)
24th week	30	96 (87–117)	34	98 (81–111)

Inter-group comparisons at baseline: * *p* = 0.548, ** *p* = 0.070, *** *p* = 0.898.

**Table 3:** Primary endpoints—Interleukin 6, Interleukin1-beta, Tumor necrosis factor-alpha.

	Placebo		Fish Oil	
	*N*	Median (IQR)	*N*	Median (IQR)
**Interleukin 6 (pg/mL)**				
Baseline *	36	1.72 (1.21, 3.01)	42	1.93 (0.02, 3.43)
12th week	36	1.31 (1.00, 2.00)	42	1.49 (0.74, 2.48)
Change ^#^	36	−0.32 (−0.89, 0.48)	42	−0.36 (−1.13, 0.52)
**Interleukin 1—Beta (pg/mL)**				
Baseline **	36	0.23 (0.05–0.52)	42	0.15 (0.02, 0.47)
12th week	36	0.25 (0.13–0.56)	42	0.17 (0.01, 0.43)
Change ^##^	36	0.00 (−0.19–0.18)	42	0.00 (−0.22, 0.24)
**Tumor necrosis factor—Alpha (pg/mL)**				
Baseline ***	36	1.05 (0.74, 1.98)	42	0.96 (0.58, 1.94)
12th week	36	0.95 (0.68, 1.47)	42	0.78 (0.55, 1.13)
Change ^###^	36	−0.06 (−0.92, 0.28)	42	−0.17 (−0.71, 0.11)

Inter-group comparisons at baseline: * *p* = 0.936, ** *p* = 0.615, *** *p* = 0.464; Inter-group changes comparisons after 12 weeks: ^#^ *p* = 0.976, ^##^ *p* = 0.884, ^###^ *p* = 0.508; IQR: interquartile range.

**Table 4:** Primary endpoints—Multilevel models.

	Coefficient	SD	z	*p*	95% Confidence Interval
**hs-CRP**					
Fish oil	0.070	0.186	0.38	0.705	−0.293 to 0.434
Time	0.009	0.049	0.17	0.861	−0.088 to 0.105
Constant	0.425	0.164	2.59	0.010	−0.104 to 0.747
Wald Chi^2^ = 0.17, *p* = 0.918					
**Fibrinogen**					
Fish oil	−0.099	0.052	−1.92	0.055	−0.200 to 0.002
Time	−0.004	0.012	−0.39	0.699	−0.029 to 0.020
Constant	4.767	0.444	129.78	0.000	5.680 to 5.854
Wald Chi^2^ = 3.82, *p* = 0.148					
**VIII factor**					
Fish oil	0.007	0.058	0.12	0.902	−0.106 to 0.120
Time	0.061	0.027	2.29	0.022	−0.009 to 0.114
Constant	4.328	0.067	64.40	0.000	−4.196 to 4.460
Wald Chi^2^ = 5.25, *p* = 0.073					

hs-CRP: high sensitivity C reactive protein.

## 4. Discussion

In the present study, a low dose of marine omega-3 fatty acids (540 mg EPA and 360 mg DHA) for 24 weeks did not change hs-CRP, fibrinogen, and factor VIII in treated HIV-infected subjects. As far as we know, this is the first study that has assessed the influence of marine omega-3 fatty acids on fibrinogen and factor VIII. Only one previous study has investigated the effects of these fatty acids on hs-CRP concentrations in a population undergoing treatment with ART [9]. Our results are consistent with previous studies in non HIV-infected populations [8,11,12].

There are four possible explanations for the absence of effects. First, the duration of supplementation with omega-3 fatty acids might have been insufficient to reverse the disturbances produced by long term exposure to HIV infection (10.6 ± 5.4 years) and ART (8.7 ± 3.9 years), and the high prevalence of smoking (42.2%) and alcohol consumption (66%). Second, the use of inflammatory markers to evaluate sub-clinical inflammation can be confounded by the susceptibility to opportunistic infection in this group. Third, a low dose of marine omega-3 fatty acids might have been insufficient to reduce inflammatory markers concentration in this population. Fourth, it is possible that the reported anti-inflammatory effects of marine omega-3 fatty acids are related to other physiological pathways rather than through a pathway involving CRP, fibrinogen, and factor VIII.

There are some limitations on this research that might have influenced our results, including the losses to follow up. Additionally, recruitment was limited by the fact that 72.0% of the 768 subjects whose records were scanned for eligibility, but were not on stable ART, and/or had high triglycerides, LDL-C concentrations, fasting glucose, and/or were taking anticoagulant, hypoglycaemic, lipid lowering drugs.

Balk *et al.* [12] reviewed the effects of omega-3 fatty acids on several cardiovascular risk factors and intermediate markers in healthy adults or those at increased risk of cardiovascular disease and did not find a consistent effect on levels of fibrinogen and factor VIII. Madsen *et al.* [8] did not detect significant reductions in hs-CRP concentrations in subjects with previous myocardial infarction after 12 weeks of supplementation with 5.2 g of omega-3 fatty acids. Thusgaard *et al.* [9] investigated the impact of 4 g of omega-3-acid ethyl esters (1520 mg DHA plus 1840 mg EPA) for 12 weeks on the inflammatory profile of HIV subjects treated with ART and described no changes in hs-CRP. Similarly, Metkus *et al.* [13] assessed the effect of 3.6 g omega-3-acid ethyl esters for eight weeks on inflammatory markers of HIV subjects and did not observe significant reduction in hs-CRP, making it unlikely that our findings were the consequence of an inadequate dose of the fatty acids. Moreover, our exclusion of data from subjects with high levels of CRP would have minimized the influence of intercurrent infection.

The mechanisms whereby marine omega-3 fatty acids might modulate inflammation include acting through a reduction and increase, respectively, of inflammatory and anti-inflammatory mediators. According to James *et al.* [14] doses of 2400 mg to 4500 mg of EPA + DHA can reduce monocyte synthesis of TNF-alpha and IL1-beta in healthy subjects and in those with rheumatoid arthritis. The ingestion of fish oil results in an elevated concentration of EPA and DHA in inflammatory cells. Consequently, there is a decrease in production of some eicosanoids such as prostaglandins E2 (PGE2), which induces the production of IL-6; and leucotriene B4 (LTB4), a chemotactic agent involved in the stimulation of macrophage production of IL-6, IL-1beta and TNF-alpha [14]. EPA is a substrate of cyclooxygenase and lipoxygenase enzymes and is converted to anti-inflammatory eicosanoids such as those of the PG3 and LT5 series [15]. Moreover, EPA and DHA are enzymatically converted into resolvins and protectins, which mediate resolution of acute phase reactions [16]. These novels mediators are involved in blocking T-cell migration and promoting apoptosis, as well as in blocking neutrophil migration, infiltration and recruitment [17]. In the trial by Thusgaard *et al.* [9] a significant rise in LTB5, an anti-inflammatory factor, was noted with supplementation of omega-3 fatty acids. Thus, further investigations should consider the assessment of more sensitive inflammatory markers to evaluate the effects of marine omega-3 fatty acids in HIV-infected subjects on ART.

## 5. Conclusions

In conclusion, a low dose of marine omega-3 fatty acids administered to HIV/Aids subjects on ART over 12 and 24 weeks did not influence levels of inflammatory markers.
